# An evolutionary conserved region (ECR) in the human dopamine receptor D4 gene supports reporter gene expression in primary cultures derived from the rat cortex

**DOI:** 10.1186/1471-2202-12-46

**Published:** 2011-05-20

**Authors:** Ursula M Paredes, Vivien J Bubb, Kate Haddley, Gabriele A Macho, John P Quinn

**Affiliations:** 1 Institute of Translational Medicine, University of Liverpool, Ashton Street, Liverpool, L69 3GE, UK; 2 MRC centre for Social, Genetic and Developmental Psychiatry Centre, Institute of Psychiatry, King's College, University of London, PO 80, DeCrespigny Park, London, SE5 8AF, UK; 3 Institut Català de Paleontologia, Campus de la Universitat Autònoma de Barcelona, 08193 Cerdanyola del Vallès, Barcelona, Spain

**Keywords:** DRD4, transcriptional, dopamine, sequence conservation, ECR, enhancer

## Abstract

**Background:**

Detecting functional variants contributing to diversity of behaviour is crucial for dissecting genetics of complex behaviours. At a molecular level, characterisation of variation in exons has been studied as they are easily identified in the current genome annotation although the functional consequences are less well understood; however, it has been difficult to prioritise regions of non-coding DNA in which genetic variation could also have significant functional consequences. Comparison of multiple vertebrate genomes has allowed the identification of non-coding evolutionary conserved regions (ECRs), in which the degree of conservation can be comparable with exonic regions suggesting functional significance.

**Results:**

We identified ECRs at the dopamine receptor D4 gene locus, an important gene for human behaviours. The most conserved non-coding ECR (D4ECR1) supported high reporter gene expression in primary cultures derived from neonate rat frontal cortex. Computer aided analysis of the sequence of the D4ECR1 indicated the potential transcription factors that could modulate its function. D4ECR1 contained multiple consensus sequences for binding the transcription factor Sp1, a factor previously implicated in DRD4 expression. Co-transfection experiments demonstrated that overexpression of Sp1 significantly decreased the activity of the D4ECR1 *in vitro*.

**Conclusion:**

Bioinformatic analysis complemented by functional analysis of the DRD4 gene locus has identified a) a strong enhancer that functions in neurons and b) a transcription factor that may modulate the function of that enhancer.

## Background

Complex behaviours are likely to be generated in part by how, where, when and by how much proteins are involved in neurotransmission. A key to understanding how such expression patterns are generated is to identify transcriptional regulatory domains for a particular gene. Comparative genetics allows the identification of such domains [1,2] and in this communication we have applied such a strategy to identify regulatory domains of the dopamine receptor D4 (DRD4). The DRD4 gene is involved in the regulation of social and cognitive phenotype of modern humans and it is preferentially expressed in the prefrontal and cingulate cortices [3-5], key centres for processing of complex behaviour and cognition. In these regions, the DRD4 protein acts as a regulator of dopamine levels, where mis-expression of DRD4 has also been associated with the onset of cognitive, behavioural and personality disorders e.g., shyness, ADHD, addiction and Parkinson's disease [6-9]. Several polymorphisms exhibiting *cis *regulatory properties have been identified in this gene. However, in spite of the interest in the DRD4 gene and its potential importance for pharmacogenetics, the regulatory machinery behind appropriate spatial-temporal gene expression remains to be elucidated.

Genome sequencing of diverse vertebrate species has permitted comparisons that reveal strong conservation of non-coding regions (evolutionary conserved regions or ECRs) between distantly related vertebrate species (e.g., between human and mouse or human and fish). Such conservation has been suggested to indicate that a given ECR could act as either a *cis *regulator of gene expression, alter post-transcriptional modifications or both [1,2]. In vertebrates ECRs have been identified in many genes involved in development [10-12] and behaviour [13].

To search for ECRs that may play a role in regulating the DRD4 gene expression, we conducted a multiple comparison of 28 vertebrate genomes at this locus using the UCSC browser (http://genome.ucsc.edu). The transcriptional activity of the most conserved ECR (D4ECR1) identified at the DRD4 locus was tested *in vitro*. Here we assessed the ability of this ECR fragment to support luciferase reporter gene expression in primary cultures of rat neonate cortical tissue. These cells were chosen for the analyses because prefrontal cortex (PFC) neurons have been reported to express the endogenous DRD4 gene during prenatal and early postnatal development in modern humans [3,4]. The expression of the DRD4 gene was also confirmed by RTPCR of cDNA extracted from tissue sections (additional file 1).

Furthermore, we assessed whether this D4ECR1 was subject to regulation by Sp1, a transcription factor which has multiple potential consensus sequences in this ECR and has been suggested as a candidate regulator for DRD4 gene expression [14]. Our data demonstrate that this bioinformatics approach is capable of identifying regulatory domains and the transcription factors that modulate them.

## Results

### Identification of an ECR in the DRD4 gene of mammals

**Figure 1:**
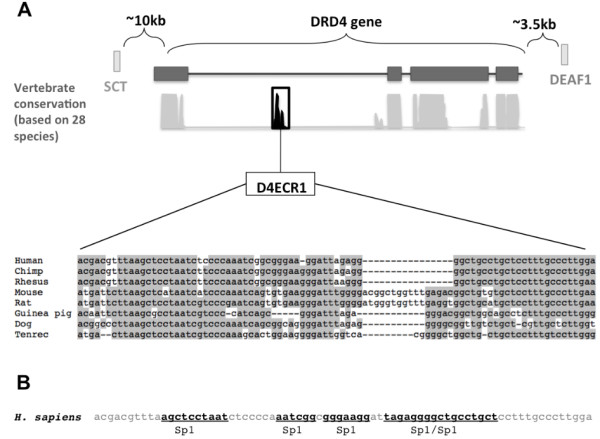
**1A. Evolutionary conserved regions (ECRs) in the DRD4 gene locus of mammals**. The conservation of non-coding regions in the DRD4 locus and flanking regions was obtained using the conservation tool of the UCSC browser, set to identify most conserved areas amongst 28 species. Neighbouring genes SCT and DEAF1 delimited the region analysed. One conserved peak (D4ECR1) in the first intron of DRD4 gene was selected for study. The alignment shows conservation of the sequence amongst 8 mammals. Dashed lines indicate no alignment. Figure 1B. Sp1 binding sites (TFBS) found in the human D4ECR1 sequence. The Alibaba 2.1 program identified 5 Sp1 sites within the sequence of the human D4ECR1 sequence (highlighted in bold font and underlined).

Examination of the DRD4 gene and flanking regions using the 28 way most conserved option of the conservation tool on the UCSC browser (limited by neighbouring genes at 10 kb upstream and 3.5 kb downstream) revealed the presence of one strong ECR in its first intron (henceforth referred to as D4ECR1, located in chr11: 628420 - 628493, 74 bp in the human genome [hg18 2006 assembly]). Location is shown in Figure 1A.

Examination of the peak of conservation generated showed that a highly homologous sequence to that of the human D4ECR1 was found in 7 other  mammalian genomes: *Pan troglodytes *(chimpanzee)*, Macaca mulatta *(rhesus macaque), *Mus musculus *(mouse)*, Rattus norvegicus *(rat), *Cavia porcellus *(guinea pig), *Echinops telfairi *(tenrec) and *Canis familiaris *(dog). Due to the lack of sequencing across the D4ECR1 locus other vertebrate genomes, it was impossible to determine if this sequence was conserved across vertebrates.

The TFBS in the mammalian D4ECR1 sequences were identified by AliBaba 2.1 (Table 1). Briefly, AliBaba is a program for predicting binding sites of transcription factors in an unknown DNA sequence; it uses the binding sites collected in TRANSFAC (4.0). This analysis identified 7 conserved binding sites for different types of TFs between the human and mouse D4ECR1 (Table 1). These TFs include: Sp1, GATA1, AP2alpha, Oct1, NF1, GR and T3Ralpha, whereby the most common binding site found in the D4ECR1 sequence was for the TF Sp1 (Figure 1B).

**Table 1:** TFBS in different mammalian D4ECR1 as identified by AliBaba 2.1.

**Species**	**Putative TFBS in the mammalian D4ECR1 sequences**
Human	Sp1, GATA1, C/EBPα, AP2α, D1, MIG1, E1, SRF, Oct1, CoS, NF1, GR, T3Rα, myogenin.
Chimpanzee	Sp1, C/EBPα, D1, E1, SRF, Oct1, CoS, NF1, GR, T3Rα, myogenin.
Rhesus macaque	Sp1, C/EBPα, D1, PU.1, FTz, E1, SRF, Oct1, CoS, NF1, GR, T3Rα.
Rat	Sp1, Oct1, YY1, RAP1, NF1, Oct1, COUP, GR.
Mouse	Sp1, GCN4, Oct1, C/EBPα, GAL4, Zen1, NF1, AP2α, GATA1, AP1, COUP, GR, T3Rα.
Guinea pig	Sp1, GATA1, NFκ, AP2α, Adf1, NF1, HNF1, GR, myogenin.
Dog	Sp1, NF1, CTF, NFκ, C/EBPα.
Tenrec	Sp1, NF1, PU.1, Fra2, C/EBPα, CRE-BP1, USF, NF1, c-Jun, MyoD, AP2α, REV-ErBAα, ER.

### Functional analysis of the human D4ECR1 demonstrated its ability to support differential luciferase expression in cultures of neonate frontal cortex

**Figure 2:**
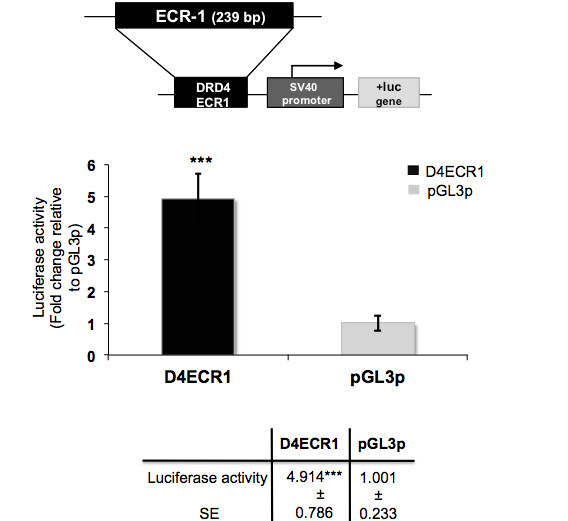
**The human D4ECR1 exhibits transcriptional activity in dissociated cultures of neonate rat frontal cortex**. D4ECR1 (1 μg) construct was transfected into dissociated cultures of frontal cortex obtained from 2 and 5 days old Wistar rats under basal conditions. The transcriptional activity of the D4ECR1 is different from pGL3p alone. This effect was found to be significant (Student's *T*-test, p < 0.001, ***). Values obtained in three independent experiments per triplicate (*n *= 9).

The potential transcriptional activity of a DNA fragment including the human D4ECR1 was validated in a reporter gene assay (Figure 2). The D4ECR1p construct was able to act as an enhancer of pGL3p reporter gene expression in primary cultures of frontal cortex obtained from 2 and 5 day old male Wistar rats. In these cell cultures, the activity supported by the D4ECR1p (4.9 fold) was significantly higher than that supported by the unmodified pGL3p control plasmid (Student's *T*-test, pGL3p vs. D4ECR1 2+5 days, p < 0.001, indicated by *** in Figure 2).

### D4ECR1 enhancer activity is regulated by overexpression of Sp1 *in vitro*

**Figure 3:**
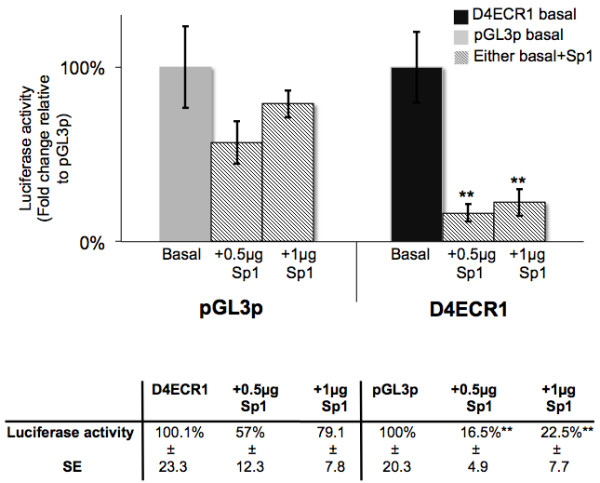
**Sp1 represses the D4ECR1 expression in dissociated cultures of neonate rat frontal cortex**. Transcriptional activities of D4ECR1 and control plasmid (pGL3p) were equalled to 100%. Both D4ECR1 and pGL3p plasmids were co-transfected with 2 concentrations of Sp1 expression vector (0.5 and 1 μg). Sp1 significantly down regulated transcriptional activity supported by D4ECR1 (Student's *T*-test p ≤ 0.01 = **; *n *= 9, 2 day old neonates). The effect of Sp1 over-expression was expressed as the percentage repression on basal transcriptional activity of D4ECR1 and pGL3p.

The identification of multiple Sp1 sites in the D4ECR1 sequence (Figure 1B) may be of importance because this TF has been previously found to bind other proposed *cis *regulators of the human DRD4 gene [14,15]. The co-transfection experiment (Figure 3) demonstrated that overexpression of Sp1 can modulate the enhancer activity of D4ECR1p in dissociated cultures of neonate rat frontal cortex at age 2 days, whereby both concentrations of Sp1 tested had a repressing effect on the transcriptional activity of the D4ECR1p.

In the graph the basal transcriptional activities of the D4ECR1p and pGL3p plasmids were normalised to 100% to express the effect of co-transfecting with Sp1 as percentages of repression. In brief, Sp1 was found to repress the activity of both the unmodified pGL3p and D4ECR1p constructs; however, this effect was only statistically significant for the D4ECR1p. (Student's *T*-test: pGL3p vs. pGL3p+ 0.5 μg of Sp1 *p *= 0.19 and vs. 1 μg of Sp1, p = 0.46). There was no significant difference in the levels of repression of D4ECR1p induced by the two concentrations of Sp1. In brief, 0.5 μg of Sp1 repressed the D4ECR1p activity in 77.5% (equal to 0.165 fold increase, *p *= 0.005 [**]) and 1 μg of Sp1 repressed D4ECR1p activity in 83.5% (equal to 0.225 fold increase, *p *= 0.003 [**]).

## Discussion

This study aimed to identify potential transcriptionally active non-coding ECRs in the DRD4 gene locus of mammals, using a comparative genomic analysis complemented and validated by reporter gene analysis. The analyses conducted demonstrated that there is at least one ECR in the DRD4 gene of mammals (Figure 1A) that exhibited transcriptional activity in frontal cortex cultures of neonate rat (Figure 2). Furthermore, in our analyses the activity of the D4ECR1p construct was reduced in these cells when the TF Sp1, predicted to bind to the D4ECR1, was over-expressed (Figure 3).

The results suggest that D4ECR1 could act as a regulator of DRD4 gene expression in the CNS. Analysis of the *in vitro *regulatory role of the human D4ECR1p construct (Figure 2) showed that it supported high levels of reporter gene expression in cultures of neonate rat frontal cortex. We observed (data not shown) that the transcriptional effects of the D4ECR1p were distinct in the cultures obtained from rats at different ages. This might be relevant as dopaminergic activity and DRD4 mRNA levels fluctuate during the first days of postnatal development in the cerebral cortex of rats [4,16-18].

DRD4 expression in the prefrontal cortex is constantly being modified *in vivo *in early postnatal development as demonstrated by previous reports (e.g. ref. [18,19]). Thus, it is possible that the D4ECR1 could be one domain that mediates such differences in regulation at this time, however there any many other regulators and domains that are likely to contribute to DRD4 expression. The variation in the activity of the D4ECR1p at these times is likely to be caused by interaction with transcription and growth factors differentially expressed during this period of development. Coincidentally, the expression of Sp1 has also been documented to fluctuate in the first days of postnatal development in the rat cerebral cortex [19].

The factors potentially binding to the D4ECR1 sequence (Table 1) indicate potential regulatory pathways operating on D4ECR1. In the present study, the TF with the highest number of binding sites found in the D4ECR1 sequence by Alibaba 2.1 was Sp1 (Figure 1B). Potential Sp1 binding sites have previously been reported in various positions within the human DRD4 locus including: the 120 bp duplication and 27 bp deletion in the 5' region [20,21]; upstream of the promoter region [22] and in the 48 bp VNTR of the 3^rd ^exon [15]. The binding of Sp1 to some of these elements has been confirmed *in vitro *by EMSA [14,15]. Furthermore, Sp1 was found to be expressed in the frontal cortex of neonate rats and has been implicated in the regulation of dopaminergic systems in the rat brain [23]. For all these reason we addressed Sp1 regulation of D4ECR1. The results indicate that the D4ECR1p described here is also responsive to Sp1 and could either individually, or in concert with the other predicted Sp1 regulated domains in the DRD4 gene modulate its expression (Figure 3). However, Sp1 is not the only transcription factor that is predicted to modulate function directed by D4ECR1 and further work is required to address how these might interact to modulate the enhancer function.

## Conclusion

The DRD4 gene harbours a non-coding ECR region in its first intron which exhibits characteristics of a *cis *regulator of gene expression in mammalian neurons. Furthermore, combination of comparative genomics and in vitro assays can be very helpful to identify novel regulatory elements in genes relevant to behavioural disorders and the transcription factors that regulate their function

## Methods

### Identification of ECRs in the DRD4 gene

The UCSC browser (http://genome.ucsc.edu/) was used to analyse the entire dopamine receptor D4 gene locus (accession number L12397) and 10000 and 3500 bases of the 5' and 3' regions respectively (extent between DRD4 and flanking genes SCT and DEAF1, Figure 1A). The browser was set to identify regions of conservation amongst the genome of 28 vertebrates using the Vertebrate Multiz Alignment & PhastCons Conservation.

### *In silico *prediction of transcription factor binding sites (TFBS) in the D4ECR1 sequence

In order to investigate which transcription factors (TFs) were potentially interacting with identified conserved regions, the sequences were subjected to *in silico *analyses, using the publicly available Alibaba 2.1 program. Alibaba 2.1 was set to detect known consensus binding sequences for TF based on the TRANSFAC 4.0 library (from the biological database webpage http://www.biobase-international.com) using the following parameters: Minimum matrix conservation (similarity between the consensus binding site for a TF and a potential binding site in the query sequence) 70%, minimum number of homologous sites (the minimum number of sites of which a matrix is build) = 4, factor class level (the classification of TFs in the TRASNFAC database is hierarchical and include 6 levels, from family of transcription factors to splice variants) = 4 and similarity of the sequence to the matrix = 1.

### Generation of the D4ECR1 reporter gene construct

*In silico *analysis of the DRD4 gene suggested presence of one strong ECR in intron 1 (termed D4ECR1). The D4ECR1 fragment identified was amplified by polymerase chain reaction (PCR) from *H. sapiens *genomic DNA (Novagen) using the following primers: DRDR4ECR1-f 5'**ggggtacccct **act cga ggt ttc ccc ttg at 3' and DRDR4ECR1-r 5' **ccgctcgagcg **tat gaa gac cgt gcc cag tg 3'. These primers were designed to encompass the D4ECR1 and incorporate flanking restriction sites for *Xho I *and *Acc65I *(bold in the primer sequences) one at either end of the primers, to facilitate cloning into the reporter gene vector pGL3p (Promega, UK), thus yielding a PCR fragment of 239bp. Briefly, conditions for PCR comprised: a reaction mix containing 100 ng of DNA template, 1 μM of each primer, 2.5 units Diamond DNA polymerase (Bioline), 1X Diamond polymerase buffer (Bioline), 0.2 mM of each dNTP, 2 mM MgCl_2_, 1 M betaine and dH_2_0 to a final volume of 50 μl per reaction. The PCR was performed for 35 cycles consisting of 55°C annealing step (1 min), 72°C extension step (1 min), and a 95°C denaturing step (1 min) in a Px2 thermal cycler (Thermo Scientific). The fragment generated by PCR was subcloned into the pGEM-T vector (Promega, UK) by TA cloning and the sequence verified. It was then released by enzymatic digestion with *Xho I *and *Acc65 I *and subsequently cloned into the multiple cloning site of the pGL3p vector, which carries a reporter gene, Firefly luciferase, driven by a minimal SV40 promoter (Promega, UK).

### Cell culture

All rats were used under local and national Schedule 1 guidelines. Primary rat frontal cortex cultures were prepared from 2 or 5 day old male Wistar rats. Briefly, frontal cortices were collected in dissection solution (HBSS [Invitrogen-Gibco] containing 100 units/ml of penicillin and 100 μg/ml of streptomycin [Sigma-Aldrich Ltd.]). The tissue was mechanically and enzymatically disassociated using 2 Pasteur pipettes of decreasing pore size in 3 ml of trypsin/EDTA solution (0.025/0.02% [Sigma-Aldrich Ltd.]). Dissociated cells were pelleted at 500 rpm and washed (3 times) with medium I (DMEM, 10% FCS, penicillin/streptomycin 100 units/ml, 100 μg/ml), resuspended and plated in poly-D-lysine (100 μl of 200 mg/ml) coated 24 well plates (5 × 10^5 ^cells per well) containing medium I (1 ml, without antibiotics) for 7 hours at 37°C in a humidified CO_2 _environment. The culture medium was changed to culture medium II (Neurobasal-A medium [Invitrogen/Gibco], 2% B27 supplement, 2 mM GlutaMAX I and 1 μg/ml of gentamycin), and cells were incubated overnight prior to transfection.

### Transfection and Co-transfection assays

The human D4ECR1-reporter gene plasmid (DRECR1p) or unmodified pGL3p vector (1 μg each) and pmLuc2 (Sea pansy luciferase expression vector [Novagen], 10 ng per 1 μg of luciferase reporter gene plasmid) were transiently co-transfected into primary cultures using ExGen 500 reagent (Fermentas) following manufacturers instructions. The pmLuc2 vector was used to control for transfection efficiency and enable standardisation of firefly luciferase values. At 48 hours post transfection, cells were lysed with 40 μl of passive lysis buffer (Promega UK, Ltd.). Firefly luciferase was quantified using the Dual Luciferase kit (Promega Ltd UK) following manufacturers instructions. Average luciferase activity values were expressed as a ratio to the reporter gene expression supported by unmodified pGL3p. Standard deviation and standard errors were calculated based on 3 independent experiments (triplicate wells).

To determine the effects of co-expression of a specific TF on the regulatory abilities of this D4ECR1, we transfected the D4ECR1p (1 μg per well) with the renillin luciferase plasmid pmLuc2 (1:100 ratio) and two different concentrations (0.5 and 1 μg) of an expression vector carrying the full length cDNA of human Sp1, kindly donated by Michael Bannon [24]. These constructs were delivered into primary cultures of neonate rat frontal cortex obtained from 2 days old rats as described above. In control experiments, the unmodified luciferase vector pGL3p (+pmLuc2) was also co-transfected with the two concentrations of Sp1 to assess possible effects of Sp1 on the backbone of the luciferase plasmid.

## Abbreviations

ECR: Evolutionary Conserved Region; DRD4: Dopamine Receptor D4; MRC: Medical Research Council; ADHD: Attention Deficit Hyperactivity Disorder; UCSC: University of California, Santa Cruz; PFC: prefrontal cortex; D4ECR1p: D4ECR1 fragment cloned into the pGL3p plasmid backbone; TFs: Transcription Factors; TFBS: Transcription Factor Binding Sites

## Authors' contributions

UMP carried out the bioinformatics, cloning and cell culture, design of experiments and drafted the manuscript. VJB contributed to cloning and drafting of the manuscript. KH contributed to cell culture, statistical analysis and drafting the manuscript. GAM revised and drafted manuscript for intellectual content. JPQ conceived of the study, and participated in its design and coordination and helped to draft the manuscript. All authors read and approved the final manuscript.

## Supplementary Material

Additional File 1 **RTPCR gel of DRD4 mRNA from rat frontal cortex**. Agarose gel showing the RTPCR products of the DRD4 receptor amplified from from sections of Wistar neonate rat brain frontal cortex. Lane 1: 1 kb ladder, lane 2: negative control, lane 3: RTPCR conducted with rat genomic DNA, lane 4: RTPCR amplification from rat brain cortex. Click here for file

## References

[B1] MacKenzie A Quinn JP Post-genomic approaches to exploring neuropeptide gene mis-expression in disease Neuropeptides 2004 38 1 15 10.1016/j.npep.2003.09.004 15003710

[B2] Wagner GP Fried C Prohaska SJ Stadler PF Divergence of conserved non-coding sequences: rate estimates and relative rate tests Mol Biol Evol 2004 21 2116 2121 10.1093/molbev/msh221 15282332

[B3] Gong S Zheng C Doughty ML Losos K Didkovsky N Schambra UB Nowak NJ Joyner A Leblanc G Hatten ME Heintz N A gene expression atlas of the central nervous system based on bacterial artificial chromosomes Nature 2003 425 917 925 10.1038/nature02033 14586460

[B4] Noain D Avale ME Wedemeyer C Calvo D Peper M Rubinstein M Identification of brain neurons expressing the dopamine D4 receptor gene using BAC transgenic mice Eur J Neurosci 2006 24 2429 2438 10.1111/j.1460-9568.2006.05148.x 17100831

[B5] Van Tol HH Bunzow JR Guan HC Sunahara RK Seeman P Niznik HB Civelli O Cloning of the gene for a human dopamine D4 receptor with high affinity for the antipsychotic clozapine Nature 1991 350 610 614 10.1038/350610a0 1840645

[B6] Arbelle S Benjamin J Golin M Kremer I Belmaker RH Ebstein RP Relation of shyness in grade school children to the genotype for the long form of the serotonin transporter promoter region polymorphism Am J Psychiatry 2003 160 671 676 10.1176/appi.ajp.160.4.671 12668354

[B7] Comings DE Gade-Andavolu R Gonzalez N Wu S Muhleman D Chen C Koh P Farwell K Blake H Dietz G MacMurray JP Lesieur HR Rugle LJ Rosenthal RJ The additive effect of neurotransmitter genes in pathological gambling Clin Genet 2001 60 107 116 11553044 10.1034/j.1399-0004.2001.600204.x

[B8] Gornick MC Addington A Shaw P Bobb AJ Sharp W Greenstein D Arepalli S Castellanos FX Rapoport JL Association of the dopamine receptor D4 (DRD4) gene 7-repeat allele with children with attention-deficit/hyperactivity disorder (ADHD): an update Am J Med Genet B Neuropsychiatr Genet 2007 144 379 382 10.1002/ajmg.b.30460 17171657

[B9] Higuchi S Muramatsu T Arai H Hayashida M Sasaki H Trojanowski JQ Polymorphisms of dopamine receptor and transporter genes and Parkinson's disease J Neural Transm Park Dis Dement Sect 1995 10 107 113 10.1007/BF02251226 9620058

[B10] Mage RG Newman BA Harindranath N Bernstein KE Becker RS Knight KL Evolutionary conservation of splice sites in sterile C mu transcripts and of immunoglobulin heavy chain (IgH) enhancer region sequences Mol Immunol 1989 26 1007 1010 10.1016/0161-5890(89)90119-3 2512480

[B11] Prabhakar S Poulin F Shoukry M Afzal V Rubin EM Couronne O Pennacchio LA Close sequence comparisons are sufficient to identify human *cis*-regulatory elements Genome Res 2006 16 855 863 10.1101/gr.4717506 16769978 PMC1484452

[B12] Shashikant CS Bolanowsky SA Anderson SM Comparison of diverged Hoxc8 early enhancer activities reveals modification of regulatory interactions at conserved *cis*-acting elements J Exp Zoolog B Mol Dev Evol 2007 308 242 249 10.1002/jez.b.21143 17171696

[B13] Davidson S Miller KA Dowell A Gildea A Mackenzie A A remote and highly conserved enhancer supports amygdala specific expression of the gene encoding the anxiogenic neuropeptide substance-P Mol Psychiatry 2006 11 4 410 421 323 10.1038/sj.mp.4001787 16402133

[B14] Ronai Z Guttman A Keszler G Sasvari-Szekely M Capillary electrophoresis study on DNA-protein complex formation in the polymorphic 5' upstream region of the dopamine D4 receptor (DRD4) gene Curr Med Chem 2004 11 1023 1029 10.2174/0929867043455503 15078164

[B15] Schoots O Van Tol HH The human dopamine D4 receptor repeat sequences modulate expression Pharmacogenomics 2003 3 343 348 *J* 10.1038/sj.tpj.6500208 14581929

[B16] Herregodts P Velkeniers B Ebinger G Michotte Y Vanhaelst L Hooghe-Peters E Development of monoaminergic neurotransmitters in fetal and postnatal rat brain: analysis by HPLC with electrochemical detection J Neurochem 1990 55 774 779 10.1111/j.1471-4159.1990.tb04559.x 1696620

[B17] Tarazi FI Baldessarini RJ Comparative postnatal development of dopamine D(1), D(2) and D(4) receptors in rat forebrain Int J Dev Neurosci 2000 18 29 37 10.1016/S0736-5748(99)00108-2 10708903

[B18] Nair VD Mishra RK Ontogenic development of dopamine D4 receptor in rat brain Brain Res Dev Brain Res 1995 90 180 183 8719342 10.1016/0165-3806(96)83499-7

[B19] Nayyar T Zawia NH Hood DB Transplacental effects of 2,3,7,8-tetrachlorodibenzo-p-dioxin on the temporal modulation of Sp1 DNA binding in the developing cerebral cortex and cerebellum Exp Toxicol Pathol 2002 53 461 468 10.1078/0940-2993-00219 11926288

[B20] Seaman MI Fisher JB Chang F Kidd KK Tandem duplication polymorphism upstream of the dopamine D4 receptor gene (DRD4) Am J Med Genet 1999 88 6 705 709 10.1002/(SICI)1096-8628(19991215)88:6<705::AID-AJMG22>3.0.CO;2-F 10581493

[B21] Szantai E Szmola R Sasvari-Szekely M Guttman A Ronai Z The polymorphic nature of the human dopamine D4 receptor gene: a comparative analysis of known variants and a novel 27 bp deletion in the promoter region BMC Genet 2005 28 6 39 10.1186/1471-2156-6-39 PMC1175085 15985158

[B22] Kamakura S Iwaki A Matsumoto M Fukumaki Y Cloning and characterization of the 5'-flanking region of the human dopamine D4 receptor gene Biochem Biophys Res Commun 1997 235 2 321 326 10.1006/bbrc.1997.6770 9199190

[B23] Zhou Q Kindlundh AM Hallberg M Nyberg F The substance P (SP) heptapeptide fragment SP1-7 alters the density of dopamine receptors in rat brain mesocorticolimbic structures during morphine withdrawal Peptides 2004 25 1951 1957 10.1016/j.peptides.2004.07.011 15501527

[B24] Wang J Bannon MJ Sp1 and Sp3 activate transcription of the human dopamine transporter gene J Neurochem 2005 93 474 482 10.1111/j.1471-4159.2005.03051.x 15816870

