# Supplementary material for: An evolutionary conserved region (ECR) in the human dopamine receptor D4 gene supports reporter gene expression in primary cultures derived from the rat cortex
Source: BMC Neurosci. 2011 May 20;12:46. doi: 10.1186/1471-2202-12-46 (PMC3121617; doi:10.1186/1471-2202-12-46)
Supplement: Additional File 1 — RTPCR gel of DRD4 mRNA from rat frontal cortex. Agarose gel showing the RTPCR products of the DRD4 receptor amplified from from sections of Wistar neonate rat brain frontal cortex. Lane 1: 1 kb ladder, lane 2: negative control, lane 3: RTPCR conducted with rat genomic DNA, lane 4: RTPCR amplification from rat brain cortex. [file 1471-2202-12-46-S1.DOC]

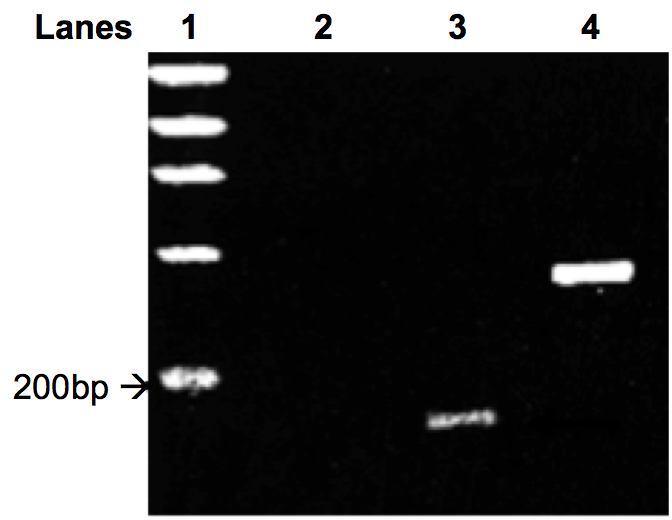


**RTPCR gel of DRD4 mRNA from rat frontal cortex.** Agarose gel showing the RTPCR products of the DRD4 receptor amplified from sections of Wistar neonate rat brain frontal cortex. Lane 1: 1kb ladder, lane 2: negative control, lane 3: RTPCR conducted with rat genomic DNA, lane 4: RTPCR amplification from rat brain cortex.
